# Energy costs of Hannibal’s alpine crossing

**DOI:** 10.1073/pnas.2612764123

**Published:** 2026-07-06

**Authors:** Emilio Berti, Fritz Vollrath

**Affiliations:** ^a^ German Centre for Integrative Biodiversity Research (iDiv) Halle-Jena-Leipzig, Theory in Biodiversity Science, Leipzig 04103, Germany; ^b^ Friedrich-Schiller University Jena, Faculty of Biological Sciences, Institute of Biodiversity, Ecology and Evolution, Jena 07743, Germany; ^c^ Department of Biology, University of Oxford, Oxford OX1 3PS, United Kingdom; ^d^ Save the Elephants, Nairobi 00200, Kenya

**Keywords:** Hannibal, alpine crossing, energy landscapes, war elephants, movement ecology

## Abstract

Hannibal crossed the Alps with a large army containing 37 war elephants in 218 BC. Details of his route have scholars regularly debate historical reports in the light of logistical and topographical considerations. Among the possible alpine passes, the route crossing at the Col du Clapier was considered the most likely candidate. Recent philological and geomorphological analyses suggest instead a route via the Col de la Traversette. Here, we examined these options focusing on the energy costs of the crossing with an emphasis on the war elephants. Our analysis favors the route of the Traversette while also providing possible insights into Hannibal’s thinking concerning both the logistics and psychology of elephant warfare.

In 218 BC the Carthaginian general Hannibal Barca crossed the Alps in 15 d with an army of 46,000 men and 37 war elephants (1) crowning a 1,000 km forced march from Spain. Without doubt, this event of the 2nd Punic war was one of the most extraordinary feats in military history. Scholars have long debated which route Hannibal followed, weighing each possibility based on historical, logistical, and topographical considerations (2). In his Histories, Polybius records his own attempt to retrace Hannibal’s route based on a contemporary account by Silenus, now lost. Polybius reports how Hannibal crossed the Rhône river and established a supply line near present-day Orange. From there, Hannibal marched North for about 80 km to Livron-sur-Drôme, from where he headed East into the Alps. After that, Polybius record is unclear, leaving it to interpretations to suggest that Hannibal chose between two possible routes across the Alps (2–5) (Fig. 1): One route passes through Grenoble and Aiton, peaks at the Col du Clapier, and reaches the Po Valley via Susa. An alternative route passes through the Col de Grimone and Gap, peaks at the Col de la Traversette, and descends into the Po Valley at Pian del Re.

**Fig. 1.**
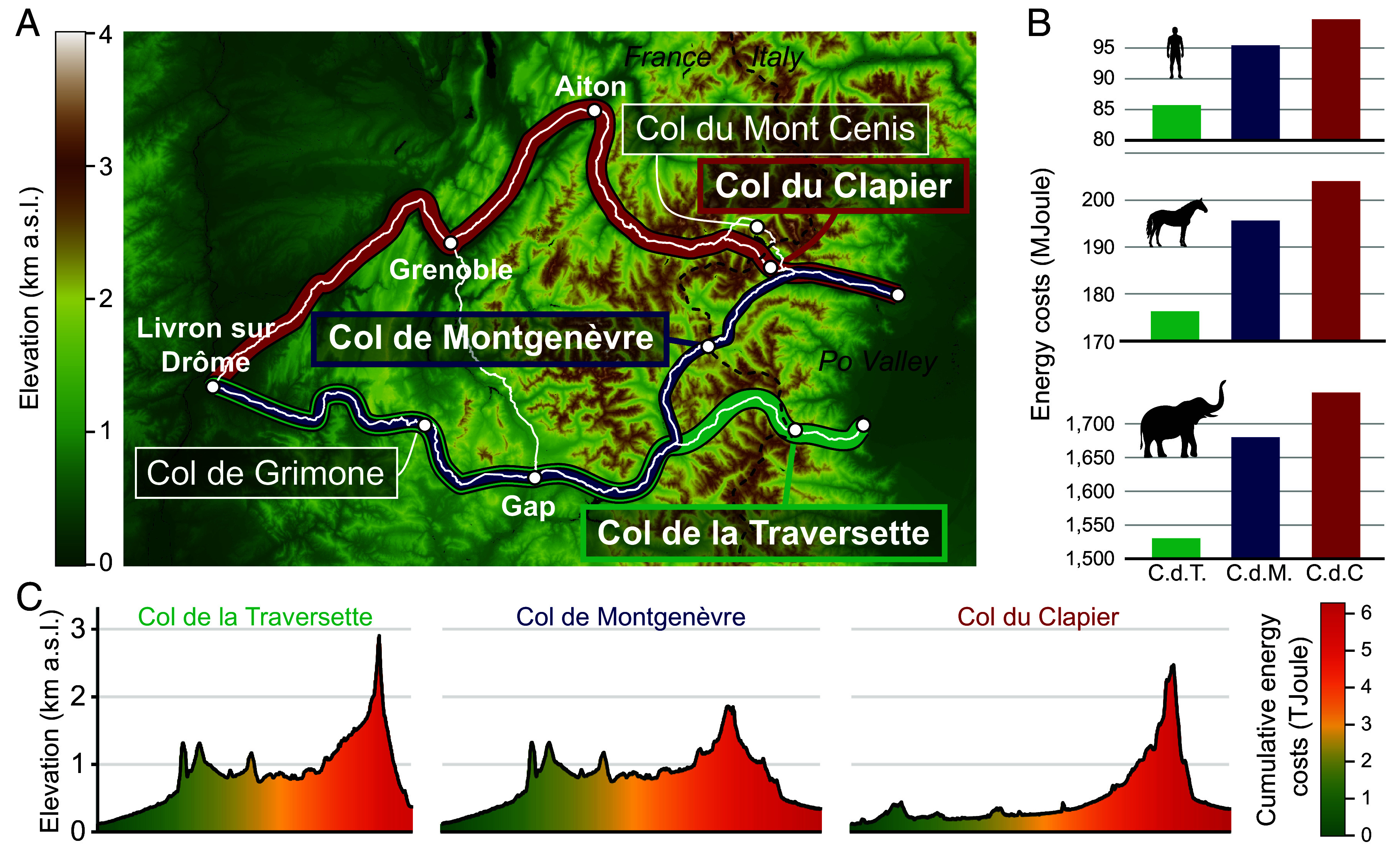
(*A*) The possible routes that Hannibal might have taken crossing the Alps. Colors show the elevation and white lines the possible routes. Colored lines show the three best routes overall. Elevation data courtesy of NASA. (*B*) Cumulative energy costs for the three overall best routes for one man, one horse, and one elephant. Colors match the routes in A. Only direct energy costs are shown, which exclude costs such as carrying food and to provide for nonmilitary personnel. (*C*) Elevation profiles of the three best routes. Colors show the cumulative energy cost for Hannibal’s army marching along the route.

Most of the discussions concerning Hannibal’s crossing were guided by philological and geological considerations, which tend to ignore the biology of the men and animals. The elephants in Hannibal’s army were large animals with enormous appetites to sustain their basic metabolic costs. Both the African elephant from the Atlas Mountains and the slightly larger Asian elephant, such as his personal ride Surus (4), weigh approximately three tons. Such weight requires considerable energy to lift (6), which is reflected in the enormous amount of forage that elephants have to source from the environment or carry along. In the wild, walking on the level without any climbing, African savannah elephants forage for about 14 h a day just to maintain their weight (6).

For any army on the march, increased energetic costs translate into greater logistical demands for food supply, increased fatigue, possible starvation, and elevated mortality among soldiers and animals. These considerations and constraints would have been especially severe for Hannibal’s army, given its size, the presence of war elephants, limited supply lines, extremely challenging terrain, and frequent hostile encounters. Only half of the men made it through the Alps, but enough war elephants survived to be deployed significantly at the battle of the Trebia (7).

## Materials and Methods

Here, we consider the conditions that Hannibal’s army and his elephants must have experienced while crossing the Alps by calculating the energetic costs for the men, horses, and elephants (8) in order to gain new insights into Hannibal’s famous endeavor. Our approach focuses on the direct energy costs of the crossing. It does not include indirect energy costs, such as foraging, carrying, and distributing food, and nor does it account for elephants’ handlers and nonmilitary personnel.

## Results and Discussion

Our analysis of the energy landscape challenging the army suggests that the Col de la Traversette would have been the shortest and energetically most efficient route, with a total cost for the whole army of 5.42 TJ (TJ = 10^12^ Joules). The route ranked second (6.02 TJ) also crossed the Col De Grimone and Gap, but then proceeded Northward and crossed the Alps at the Col de Montgenèvre and reached the Po Valley from Susa. The Col du Clapier route was ranked the third (6.28 TJ). Finally, the route crossing Col du Mont Cenis was the least efficient option (6.45 TJ). Compared to choosing the Col de la Traversette, the routes via the Col de Montgenèvre, Col du Clapier, and Col du Mont Cenis would have required 11%, 16%, and 19% more energy, respectively, for the army as a whole. The proportions were comparable when considering only the elephants, the horses, and the men (Table 1). Although Hannibal would not have had such accurate estimates, he may have had a qualitative understanding of the ranking of the possible routes. In which case, driven by the aim to minimize the energy costs of the crossing, he would have chosen the Traversette route.

**Table 1.** Energy costs and speed calculated for the different routes crossing the Alps

Route	Army (10^12^ J)	Man (10^6^ J)	Horse (10^6^ J)	Elephant (10^9^ J)	Speed (km/day)
Col de la Traversette	5.42	86.01	176.43	1.53	25
Col de Montgenèvre	6.02 (11%)	95.53 (11%)	195.71 (11%)	1.68 (10%)	29
Col du Clapier	6.28 (16%)	99.67 (16%)	204.07 (16%)	1.75 (14%)	30
Col du Mont Cenis	6.45 (19%)	102.27 (19%)	209.68 (19%)	1.79 (17%)	31

Joules (J) are standard units of energy and are defined as kg m^2^ s^−2^.

Speed was calculated dividing the total length of the routes by 15 d, which was the total duration of the crossing (1).

Percentages show the amount of extra energy needed compared to crossing at Col de la Traversette.

Assuming carbohydrates as the main food source for men (17 KJ/g), the energy costs for crossing at the Col de la Traversette translated into 232.72 tons of supplies needed, which increased by 25.76, 36.98, and 44.00 tons for the three other routes, respectively. While the men, and perhaps the horses, could have carried each their own provisions, this would have been impossible for the elephants, as a three-ton adult would normally consume per day ~200 kg of forage in the wild and ~75 kg of feed in captivity just to satisfy basal metabolic demands. Thus, assuming an average daily basal metabolic demand of 119 MJ/day (9) and a reported travel time of 15 d (1), the total basal metabolic costs, without any climbing and descending, would have been 1.79 GJ. Crossing at the Col de la Traversette would have cost each elephant an additional 1.53 GJ of energy. For the other three routes, this would have increased by an additional 1.68 GJ, 1.75 GJ, and 1.79 GJ, respectively. In order to keep body weight constant, elephants typically need about one hour of foraging to replenish an energy expenditure of 20 MJ (6). Hence, for the elephants to have kept their body weight during the crossing, they would have required 5 to 6 h of extra feeding each day, i.e. near-continuous feeding. This scenario is highly unlikely and so we must assume that Hannibal’s elephants would have had to rely on their body fat reserves for the duration of the crossing.

To assess how severely limited food intake would deplete energy reserves in the animals and men, we calculated (10) how much of their body fat reserves elephants, horses, and men would have consumed during the crossing. Accordingly, when crossing the Col de la Traversette, elephants would have lost 4% of their body fat reserves, horses 11%, and men 19%. These values would not have substantially increased for the elephants concerning the other three routes but would have increased between 12% and 13% for horses, and between 21% and 22% for the men. Importantly, elephants and men would have faced rather different challenges. Thanks to their enormous body fat storage, elephants would have crossed the Alps using only a small fraction of it. Once arrived in Italy, however, it would have required significant time and fodder to replenish these energy reserves and to keep the elephants alive during the cold and wet winter and considerably more still to fit them for subsequent marches and battles. Although the men who survived the crossing would have lost a relatively large proportion of their fat reserves, these would have been replenished faster and more effectively. In any case, having crossed the Alps, what remained of Hannibal’s army would have found itself isolated in enemy territory without supply lines. Evidence suggests that the Italian farmland (and perhaps also riparian woodlands) were extensively raided to meet the demands of all its men and animals (1). The energetic situation of Hannibal’s army would have been critical already just after crossing the Alps.

Hannibal managed to get many, if not most, elephants across the Alps, with around 30 estimated to have fought at the Battle of the Trebia (7), although all but Surus died the following winter. The astonishing feat of crossing the Alps with elephants has been a puzzle ever since: Not just “how” but also “why” even attempt to cross the Alps with elephants? We may assume that Hannibal knew, after having traveled with elephants and their handlers through Spain and Southern France, that his elephants would struggle less than his men to make the crossing. Indeed, in addition to having large fat energy reserves, we now know that elephants move akin to a four-wheel-drive vehicle (11). This would make them particularly suitable for mountaineering, as more recent accounts also confirm (12). Perhaps Hannibal would have been more concerned with what to do with the elephants once in Italy, as replenishing their fat reserves and keeping them alive would have been hugely challenging, isolated as he was in enemy territory without supply lines. Perhaps he counted on the elephants to provide an important, tactical element of surprise in the first battles against the Romans. In addition, he may have expected that his war elephants would awe and help recruit to his side the Celts of Northern Italy who were known to be hostile to Rome. After these first encounters, the elephants might have lost their military value. The fact that all but one elephant died in the following winter might be indicative of Hannibal’s decision to stop attending to the energy needs of such costly pieces of military equipment when already under severe energetic constraints.

After crossing the Alps, Hannibal waged war in Italy for 14 y, defeating the Romans in major set-piece battles. Despite military success, Hannibal could not force Rome to surrender and returned to Carthage. In 201 BC, an invading Roman force defeated the Carthaginian army fielding 80 war elephants and lead by Hannibal at Zama. Carthage surrendered, which concluded the second Punic War. The hostilities between Rome and Carthage started once more in 149 BC, this time ending with the destruction of Carthage and the extermination and enslavement of its population in 146 BC.

## Supplementary Material

Appendix 01 (PDF)

## Data Availability

GIS layers and code data have been deposited in Zenodo (13).
