# Supplementary material for: Energy costs of Hannibal’s alpine crossing
Source: Proc Natl Acad Sci U S A. 2026 Jul 6;123(28):e2612764123. doi: 10.1073/pnas.2612764123 (PMC13367890; doi:10.1073/pnas.2612764123)
Supplement: Supplementary file 1 — Appendix 01 (PDF) [file pnas.2612764123.sapp.pdf]

PNAS

Supporting Information for
Energy costs of Hannibal's alpine crossing

Emilio Berti[a,b,1] and Fritz Vollrath[c,d]

[a] German Centre for Integrative Biodiversity Research (iDiv) Halle-Jena-Leipzig, Theory in Biodiversity Science, Leipzig 04103, Germany

[b] Friedrich-Schiller University Jena, Faculty of Biological Sciences, Institute of Biodiversity, Ecology and Evolution, Jena 07743, Germany

[c] Department of Biology, University of Oxford, Oxford OX1 3PS, United Kingdom

[d] Save the Elephants, Nairobi 00200, Kenya

[1] To whom correspondence may be addressed. Email: emilio.berti@idiv.de.

This PDF file includes:

Supporting text
SI References

Supporting Information Text

Extended Methods

Reconstructing the routes. We reconstructed the potential routes that Hannibal could have used to cross the Alps based on previous work (1-4). We refer here and in the main text to the current names of the locations. The common origin of all this routes is likely located near Livron-sur-Drôme, south of Valence, France. From here, Hannibal likely marched either to Grenoble or, through the Col de Grimone, to Gap. From Grenoble, Hannibal could have either proceeded South, reaching Gap, or East, passing through Aiton and crossing the Alps either at the Col du Mont Cenis or at the Col du Clapier, and reached the Po Valley through Susa, West of Turin. From Gap, Hannibal could have either marched North and reach Susa passing through the Col du Montgenèvre, or marched East and crossed the Alps at the Col de la Traversette, reaching the Po Valley through Pian del Re.

Instead of focusing on a whole route, we build a spatial graph *G(V, E)* with the locations mentioned above and in the figure in the main text as vertices (nodes) and the paths shown in as edges (links). The edges were obtained as spatial geometries from Google Map using the Google My Map application (https://www.google.com/maps/d/). Because roads and walking paths are usually build following low-cost routes, we assumed that the routes obtained using Google Map to be representative of the potential routes that an army would follow. We inspected these routes visually and adjusted them in case of visible discrepancy between the proposed route and a clearly more optimal one. For example, it happened that a route crossed a peak that could have been avoided by traveling through a close valley. We first tried to switching from "walking" to "driving mode", as some roads can only be used by vehicles or on foot. If this did solve the issue, we adjusted the route manually. We drew manually a straight line of circa 800 meters between the Col de la Traversette and the trail leading to Pian del Re. We exported all routes as shapefiles and combined the segments to obtain the edges *E* of the graph *G*. We then obtained all possible routes that Hannibal would have used using the *all_simple_paths()* function from the R package *igraph* (5), specifying the origin at Livron-sur-Drôme and the end in the Po Valley. Note that, despite the location "Po Valley" was geographically in two distinct locations, these were the same vertex in the graph.

Cost of the routes. We calculated the costs of traveling along each route using the R package *enerscape* v1.2.0 (6), specifying the body mass of elephants equal to 3,160 kg, for horses to 200 kg, and for men to 70 kg. The biomechanical model to calculate costs used in enerscape was a model shown to work for a general legged tetrapod animal (7) for elephants and horses, while a model specifically tailored to humans was used for men (8). We used the digital elevation model (DEM) from NASA at 30 meter resolution to calculate terrain slopes and energy costs (9). First, we extracted the elevation profile of each route. Second, in order to remove spurious records of elevation due to unaccounted landscape feature, such as water bodies and trees, we smoothed the DEM by calculating the average within a rolling window after excluding outliers (elevation spikes). Specifically, for each step i, we extracted the elevation values in the window $\boldsymbol{x}=[x_{i-10}, x_{i+10}]$, calculated the median of $\boldsymbol{x}$ and its median absolute deviation (MAD), removed values that were outside the median ± 2 MAD, and reassigned to x_i the average of the remaining values in $\boldsymbol{x}$. To calculate the total costs for the whole army, we used the numbers reported by Polybius before the ascent of the Alps begun: 37 elephants, 8,000 war horses, and 46,000 foot soldiers, including 8,000 cavalrymen that likely crossed the Alps on foot. All geographic analyses were conducted in the projected system UTM32N (EPSG code 3064).

Depletion of energy reserves. We calculated how much energy reserves, stored in the form of body fat, animals and men would lose during the crossing using a scaling relationship between body mass and body mass fat (10). Specifically, the body mass fat (M_{fat}, kg) scales with body mass (M, kg) following

$$M_{fat} = 0.075 M^{1.19} \tag{1}$$

Given a fat to energy conversion of 39,300 J/kg (10), we calculated how much body fat mass each individual elephant, horse, and man would lose, on average, for each possible route. Given a total energy cost for a route of E_i (J), the loss of body fat mass (ΔM_{fat}, kg) was calculated as

$$\Delta M_{fat} = \frac{E_i}{39300} \quad (2)$$

We also calculated the proportional loss of body mass fat and total mass:

$$L_{fat} = \frac{\Delta M_{fat}}{M_{fat}} \quad (3)$$

$$L = \frac{\Delta M_{fat}}{M} \quad (4)$$

Where L_{fat} is the proportion of fat body mass loss and L the proportion of total mass loss.

SI References

1. W. Bainbridge, S. Cracolici, Tall talk about elephants: Hannibal's crossing through disciplines. Hist Res htaf020 (2025). https://doi.org/10.1093/hisres/htaf020.
2. G. De Beer, Hannibal: the struggle for power in the Mediterranean (Thames & Hudson, 1969).
3. W. C. Mahaney, Polybius: decipherer of Hannibal's alpine route and ancient stratigrapher. Int J Earth Sci (Geol Rundsch) 112, 1989–1995 (2023).
4. M. Kuhle, S. Kuhle, Lost in Translation or Can We Still Understand What Polybius Says about Hannibal's Crossing of the Alps?—A Reply to Mahaney [Archaeometry, 55 (2013), 1196–204]*. Archaeometry 57, 759–771 (2015).
5. G. Csardi, T. Nepusz, The igraph software package for complex network research.
6. E. Berti, et al., The r package enerscape: A general energy landscape framework for terrestrial movement ecology. Methods in Ecology and Evolution 13, 60–67 (2022).
7. H. Pontzer, A unified theory for the energy cost of legged locomotion. Biology Letters 12, 20150935 (2016).
8. D. P. Looney, et al., Estimating Energy Expenditure during Level, Uphill, and Downhill Walking. Medicine & Science in Sports & Exercise 51, 1954–1960 (2019).
9. NASA JPL, NASADEM Merged DEM Global 1 arc second V001. NASA EOSDIS Land Processes DAAC. https://doi.org/10.5067/MEASURES/NASADEM/NASADEM_HGT.001. Deposited 2020.
10. S. L. Lindstedt, M. S. Boyce, Seasonality, Fasting Endurance, and Body Size in Mammals. The American Naturalist 125, 873–878 (1985).
